# Cholesteryl Esters Accumulate in the Heart in a Porcine Model of Ischemia and Reperfusion

**DOI:** 10.1371/journal.pone.0061942

**Published:** 2013-04-26

**Authors:** Christina Drevinge, Lars O. Karlsson, Marcus Ståhlman, Thomas Larsson, Jeanna Perman Sundelin, Lars Grip, Linda Andersson, Jan Borén, Malin C. Levin

**Affiliations:** 1 Wallenberg Laboratory, Sahlgrenska University Hospital, Göteborg, Sweden; 2 Department of Molecular and Clinical Medicine, Institute of Medicine, Sahlgrenska Academy at University of Gothenburg, Göteborg, Sweden; Virginia Commonwealth University, United States of America

## Abstract

Myocardial ischemia is associated with intracellular accumulation of lipids and increased depots of myocardial lipids are linked to decreased heart function. Despite investigations in cell culture and animal models, there is little data available on where in the heart the lipids accumulate after myocardial ischemia and which lipid species that accumulate. The aim of this study was to investigate derangements of lipid metabolism that are associated with myocardial ischemia in a porcine model of ischemia and reperfusion. The large pig heart enables the separation of the infarct area with irreversible injury from the area at risk with reversible injury and the unaffected control area. The surviving myocardium bordering the infarct is exposed to mild ischemia and is stressed, but remains viable. We found that cholesteryl esters accumulated in the infarct area as well as in the bordering myocardium. In addition, we found that expression of the low density lipoprotein receptor (LDLr) and the low density lipoprotein receptor-related protein 1 (LRP1) was up-regulated, suggesting that choleteryl ester uptake is mediated via these receptors. Furthermore, we found increased ceramide accumulation, inflammation and endoplasmatic reticulum (ER) stress in the infarcted area of the pig heart. In addition, we found increased levels of inflammation and ER stress in the myocardium bordering the infarct area. Our results indicate that lipid accumulation in the heart is one of the metabolic derangements remaining after ischemia, even in the myocardium bordering the infarct area. Normalizing lipid levels in the myocardium after ischemia would likely improve myocardial function and should therefore be considered as a target for treatment.

## Introduction

Myocardial infarction/ischemia is a major cause of death worldwide and survivors from a myocardial infarction remain at high risk of death in the years after the event. Myocardial ischemia results in irreversible cardiac damage with subsequent cell death, and in addition causes changes in the metabolic and functional characteristics of surviving cells. At baseline, the heart uses a diverse set of substrates for energy metabolism, such as fatty acids, lactate and glucose. [1], [2] Studies in cell and rodent models have shown significant alterations in myocardial substrate utilization after ischemia. Indeed, myocardial ischemia in mice is associated with accumulation of lipids. [3]

Lipid accumulation in the heart correlates with reduced heart function. [4], [5] Although ischemia-induced lipid accumulation can be explained in part by decreased fatty acid oxidation, we have recently shown that myocardial ischemia actually induces an increased uptake of exogenous lipids. [3] This increased uptake of lipids was induced by endocytosis of triglyceride rich lipoproteins mediated through the very low density lipoprotein receptor (VLDLr). We found that Vldlr^−/−^ mice showed improved survival and decreased infarct area following an induced myocardial infarction. Furthermore, ischemia-induced ER stress and apoptosis in mouse hearts were reduced in Vldlr^−/−^ mice. [3]

Lipid loading in the myocardium during pathological conditions such as myocardial ischemia may lead to bioactive lipid metabolites (e.g. sphingolipids such as ceramides) accumulating under these conditions and cause cellular dysfunction. Ceramides may be synthesized de novo by up-regulation of the sphingolipid synthesis pathway or by breakdown from sphingomyelin. [6] Proposed mechanisms for the link between increased intracellular lipid content and reduced heart function include apoptosis, inflammation and/or endoplasmatic reticulum (ER) stress. [7], [8]

Substrate alterations, and subsequent lipid accumulation, in the ischemic heart are potential targets of therapeutic modulation in the failing myocardium. Thus, it is of major importance to use a model system of physiological significance. The pig has a cardiovascular system that resembles that of humans, both in terms of cardiac physiology and collateral flow. [9], [10] In this study, we therefore investigated derangements of lipid metabolism that are associated with myocardial ischemia in a porcine model of ischemia and reperfusion.

## Materials and Methods

After approval from the Gothenburg ethics committee for animal experiments (permit number 379–2008) at Gothenburg University, animal experiments were performed in accordance with the European Convention for the Protection of Vertebrate Animals used for Experimental and other Scientific Purposes (Council of Europe No 123, Strasbourg 1985).

### Ischemia and reperfusion procedure

Seven female pigs of a mixed Swedish, Pigham and Yorkshire race (weighing 38–46 kg, 3–4 months old) were used for a well established closed-chest model of ischemia and reperfusion. [11], [12] The pigs were bred in Swedish farms and brought to the animal laboratory one week prior to the ischemia and reperfusion procedure, where they were kept together until the intervention. The day before the procedure the pigs received 75 mg of acetic-salicylic acid (Trombyl, Pfizer, Sollentuna, Sweden). The animals were sedated with intramuscular tiletamin and zolazepam (Zoletil, Virbac, Carros, France), 6 mg/kg each, suspended in Dexdomitor (Orion Pharma, Esbo, Finland), and 0.5 mg atropine (Mylan AB, Stockholm, Sweden) was administered intramuscularly. General anaesthesia was induced by intravenous injection of 3–10 mg/kg 2% thiopental (Pentobarbital, Apoteksbolaget, Stockholm, Sweden), and mechanical ventilation with 40% oxygen was performed via oral intubation. Anaesthesia was maintained by a continuous intravenous infusion (4–18 mg/kg/h) of thiopental. Analgesia was assured by continuous intravenous infusion of morphine (MEDA AB, Stockholm, Sweden) (0.5 mg/kg/h). Venous access was established by a 5 French introducer sheath through the right jugular internal vein. Monitoring of mean arterial pressure and heart rate was achieved through an arterial cannulae in the femoral artery. Respiratory rate and tidal volume were adjusted to keep arterial blood pH, pO2 and pCO2 within the physiological range. Endotracheal temperature was maintained at 37.5–39°C, if necessary through external heating. Blood pressure was supported by infusion of plasma expanding solutions (Ringer-Acetat, Baxter Medical AB, Kista, Sweden).

At the commencement of catheterization all pigs received 200 units/kg of unfractionated heparin (Leo, Ballerup, Denmark) which was repeated with 100 units/kg every hour. After 60 min of stabilization, a 6 Fr JR 3.5 PCI-guiding catheter was inserted via an 8 Fr introducer sheath into the right carotid artery, and under X-ray guidance (OEC 9800 Cardiac™ General Electric Medical Inc., UT, USA) placed in the left coronary ostium. An angiogram with contrast agent Visipaque® (320 mg/ml) (Nycomed-Amersham, Oslo, Norway) was performed and an angioplasty balloon inflated (2.0–3.0 mm in diameter, 10–20 mm long, 8–14 atmospheres, respectively) in the left anterior descending artery distal to the second diagonal branch. Occlusion was verified angiographically and through ST-segment alteration on the ECG. After 40 min of occlusion the balloon was deflated and reperfusion verified by contrast injection.

After four hours of reperfusion the chest was opened to obtain access to the heart and great vessels. Another coronary angiogram was performed, an angioplasty balloon inflated at the same location as before and a small suture placed around the balloon. 40 mL of 2% Evans blue was infused via the central venous catheter, followed by an injection of a lethal dose of potassium chloride. The heart was excised and the right ventricle and the atria removed. The left ventricle was sliced transversely into 10 mm thick sections and photographed. The area not stained with Evans blue constituted the ischemic area whereas the stained tissue represented the control area (not subjected to ischemia).

Myocardial biopsies from the free lateral wall (control area), the lateral border of the ischemic area (area at risk, AAR, presumed not to be infarcted) and the central, subendocarial aspect of the ischemic area (infarct area, IA, presumed to be infarcted) were immediately frozen in liquid nitrogen and stored at −70°C until further analysis. The slices were thereafter incubated in 2, 3, 5 triphenyltetrazolium chloride for 10 minutes at 37°C in order to delineate the IA, and once again photographed. By this means we could confirm that the myocardial biopsies had been taken from the control area, AAR and IA, respectively.

### Measurements of lipid droplet area

To determine the size of the total lipid droplet area, frozen pig heart biopsis were cryosectioned in 8 µm sections and transferred to SuperFrost Plus glass slides (Menzel-Gläser). The sections were fixed in Histofix (HistoLab Products AB) for 5 minutes, pretreated with 20% isopropanol for 30 seconds, and stained with Oil Red O in 60% isopropanol for 15 minutes. The cells were washed with 20% isopropanol for 30 seconds and then washed in cold water. The coverslips were mounted on microscope slides with Mowiol (Calbiochem, Darmstadt, Germany) and viewed with an ApoTome Axioplan 2 imaging system (Carl Zeiss, Göttingen, Germany). Images were obtained with an Axiocam camera and Axiovision 4.7 software. The total Oil Red O–stained surface area was quantified in 20 fields per section, using the image analysis software BioPix to analyze the neutral lipid area that is stained red, as previously described. [13]

### Analysis of gene expression

Total RNA was extracted from homogenised heart tissue using the Rneasy fibrous tissue mini kit (Qiagen). cDNA was synthesized using the high capacity cDNA Reverse Transcription Kit (Applied Biosystems, Foster City, CA) with random primers. mRNA expression of genes of interest was analyzed with TaqMan real-time PCR in an ABI Prism 7900 HT Detection System (Applied Biosystems) and normalized to the reference gene 18s. The following TaqMan Gene Expression assays from Applied Biosystems were used: interleukin (IL)-1β Ss03393804_m1, IL-6 Ss03384604_u1, CCAAT/-enhancer-binding protein homologous protein (CHOP) Ss03821509_s1, Activating transcription factor 6 beta (ATF6b) Ss03390340_m1, Low density lipoprotein receptor (LDLr) Ss03374441_u1, Very low density lipoprotein receptor (VLDLr) Ss03374049_m1, Scavenger receptor class B member 1 (SR-B1) Ss03391104_m1, and 18 s 4319413E.

### Immunoblot

Proteins were extracted from frozen homogenized tissue using the Qproteosome Mammalian Protein Prep Kit (Qiagen). Equal amounts of total protein were loaded onto a NuPAGE 4–12% Bis-Tris Gel (Novex, Invitrogen, Carlsbad, CA). The proteins were transferred to nitrocellulose membranes and incubated with antibodies that recognized LDLr ab30532 (Abcam), Low density lipoprotein receptor-related protein 1 (LRP1) 2703-1 (Epitomics), Calnexin #2679 (Cell Signaling), 78 kDa glucose-regulated protein (GRP-78) 610979 (BD Biosciences) or Beta tubulin ab6046 (Abcam). Proteins were visualized with horseradish peroxidase-conjugated secondary antibodies (Amersham Biosciences) and Immobilon Western (Millipore). Bands were quantified with Multi Gauge V3.0 (Fujifilm Life Sciences) and normalized to Beta tubulin.

### Hypoxia incubation of HL-1 cells

The HL-1 cardiomyocyte cell line was cultured as described previously [14] and incubated in supplemented Claycomb media [14] at 21% oxygen (normoxia) or 1% oxygen (hypoxia) for 0.5, 1, 6, or 24 hours.

### Lipid class fractionation and quantification by HPLC

Lipids were extracted as using the Folch method. [15] Heptadecanoyl (C17∶0)-containing phospholipids, sphingomyelin and ceramide was diluted in chloroform and added during the extraction procedure. Total lipid extracts were reconstituted in heptane-isopropanol (9∶1; v/v) and separated using straight-phase HPLC as previously described. [16] Post-column, the flow was split so that 25% of the flow (400 µl/min) was directed into a PL-ELS light scattering detector (Polymer Laboratories, Amherst, MA, USA). This detector was used for quantification of cholesteryl esters, triacylglycerols and free cholesterol. The other 75% (1200 µl/min) was directed to a Gilson FC 204 fraction collector (Gilson, Middleton, WI, USA). The ceramides were collected for further analysis by mass spectrometry.

### Lipid analysis using mass spectrometry

The phospholipids phosphatidylcholine (PC) and phosphatidylethanolamine (PE) and sphingomyelin was analyzed using shotgun analysis directly from a total extract as described previously. [17] An aliquot of the total lipid extract was evaporated and re-dissolved in chloroform-methanol (1∶2 v/v) with 5 mmol/l ammonium acetate. The analysis was made using precursor ion scanning in positive and negative mode on a QTRAP 5500 mass spectrometer (MDS Sciex, Concord, Canada) equipped with a robotic nanoflow ion source, TriVersa NanoMate (Advion BioSciences, Ithaca, NJ. Quantification was made using the heptadecanoyl (C17∶0)-containing standard added during the lipid extraction. After purification using straight-phase HPLC, the ceramides were analyzed using reversed phase HPLC coupled to a triple quadrupole Quattro Premiere mass spectrometer (Waters, Milford, USA) as previously described. [18] Quantification was made using a combination of external standards.

### Statistical analysis

Measurements were compared with 1-way ANOVA followed by Dunnetts post hoc test. P-values <0.05 were considered significant. Data are shown as means ± SEM.

## Results

### Myocardial ischemia promotes accumulation of lipid droplets

**Figure 1:**
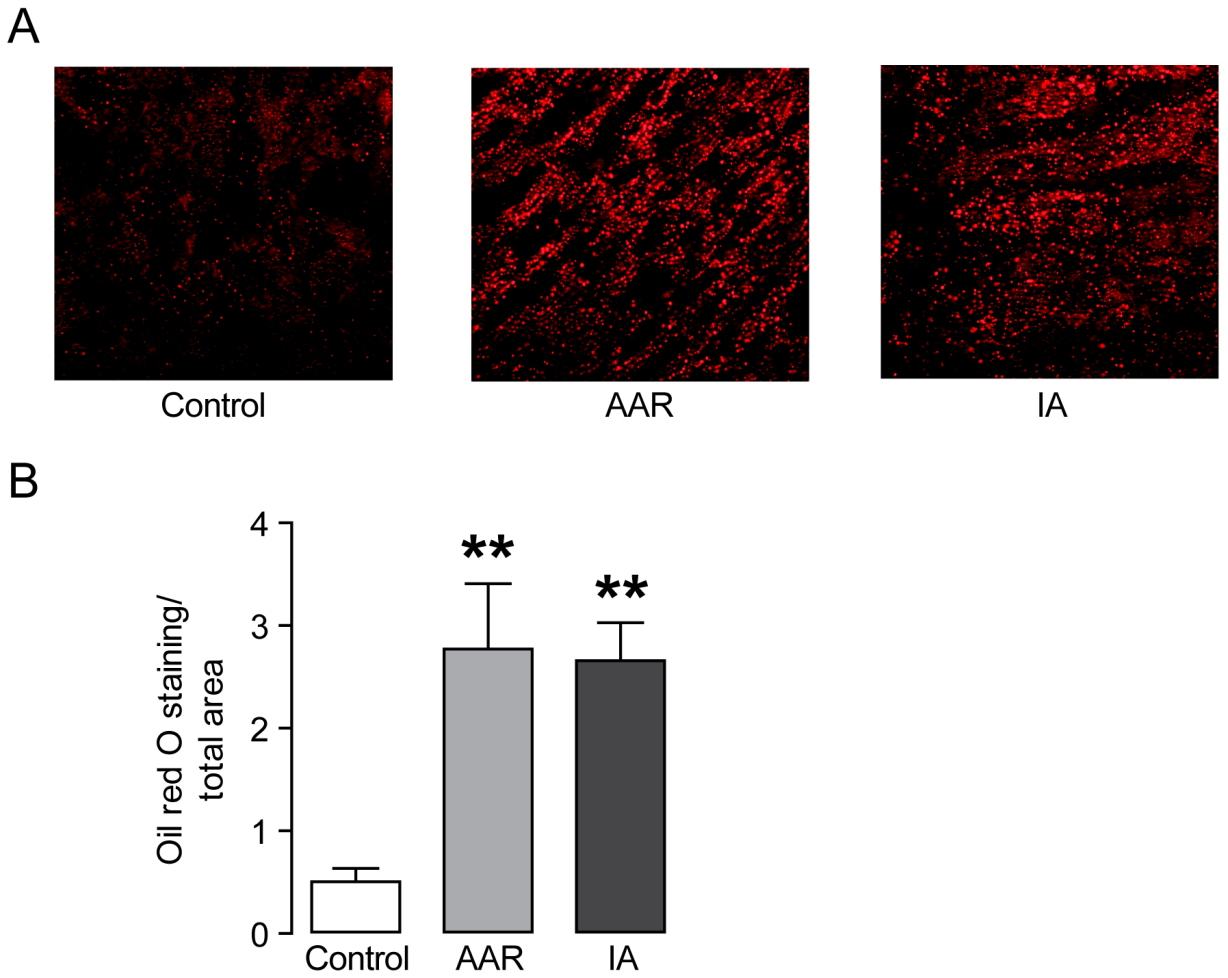
Increased accumulation of lipid droplets in the AAR and IA. **(A)** Representative images of Oil red O stained cryosections. **(B)** Quantification of the total Oil red O stained area. Results are shown as mean ± SEM, **P<0.01 *vs.* control.

To investigate where lipids accumulate in the pig heart following ischemia/reperfusion, we performed an Oil red O staining of cryosections from heart biopsies. The large pig heart enables the separation of the infarct area (IA) with irreversible injury from the area at risk (AAR) with reversible injury and the unaffected control area. The surviving myocardium bordering the infarct (i.e. the AAR) is exposed to mild ischemia and is stressed, but remains viable. Quantification of the Oil red O stained surface area showed significantly increased accumulation of lipid droplets in the AAR as well as the IA compared to the control area (Figure 1A–B).

### Accumulation of cholesteryl esters in the ischemic porcine heart

**Figure 2:**
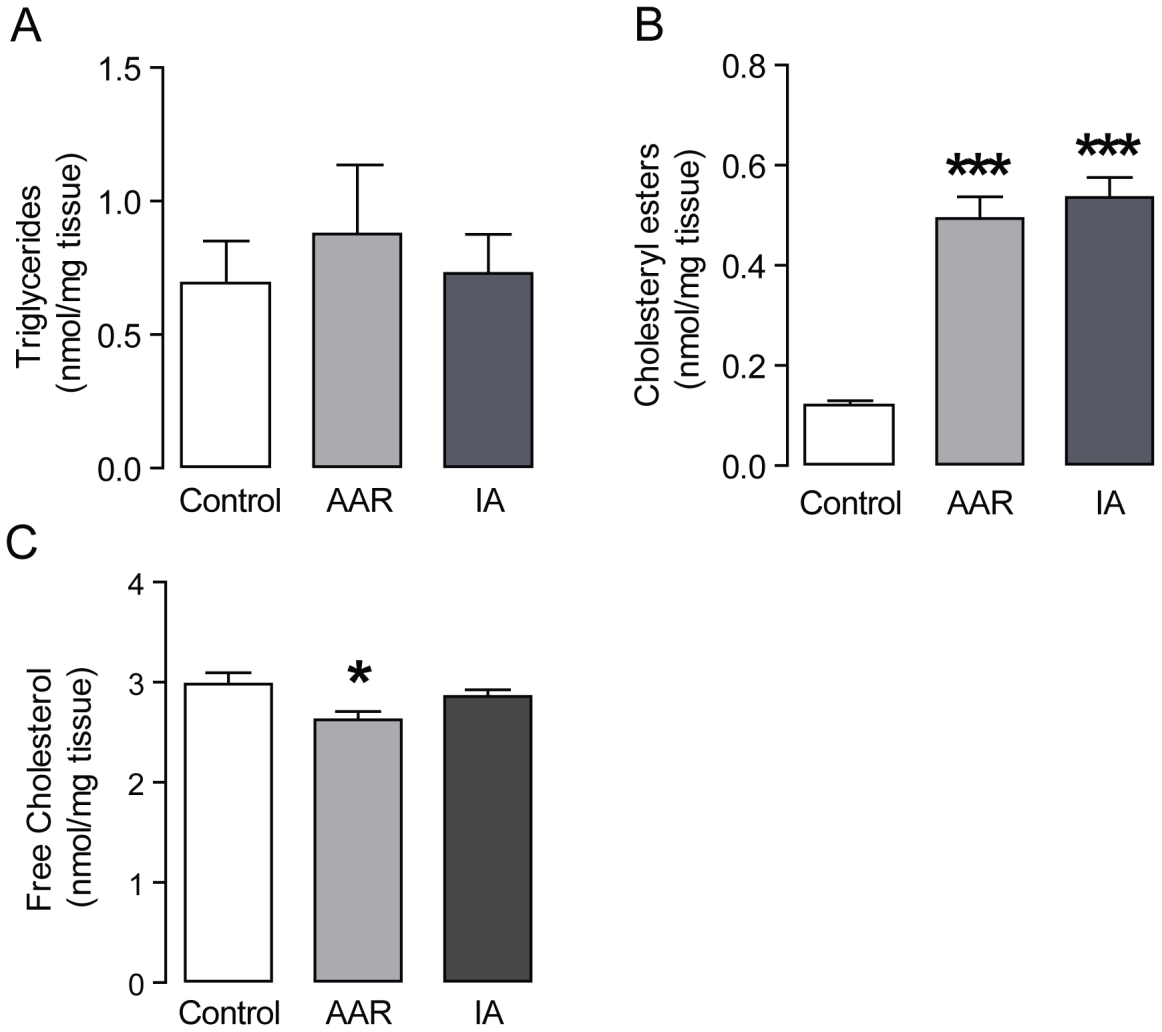
Increased levels of cholesteryl esters in the pig myocardium. **(A)** Content of cholesteryl esters, **(B)** triglycerides and **(C)** free cholesterol, n = 7 per group. Results are shown as mean ± SEM, ***P<0.001 *vs.* control.

To further investigate lipid accumulation, we measured the content of triglycerides, cholesteryl esters and free cholesterol. Interestingly, we found that triglyceride levels were not affected in the AAR or IA (Figure 2A). However, cholesteryl esters were greatly increased in the AAR as well as the IA (Figure 2B). The free cholesterol levels were slightly decreased in AAR, but not affected in the IA (Figure 2C).

### Low Density Lipoprotein receptor (LDLr) and low density lipoprotein receptor-related protein 1 (LRP1) expression is increased in the ischemic porcine heart

**Figure 3:**
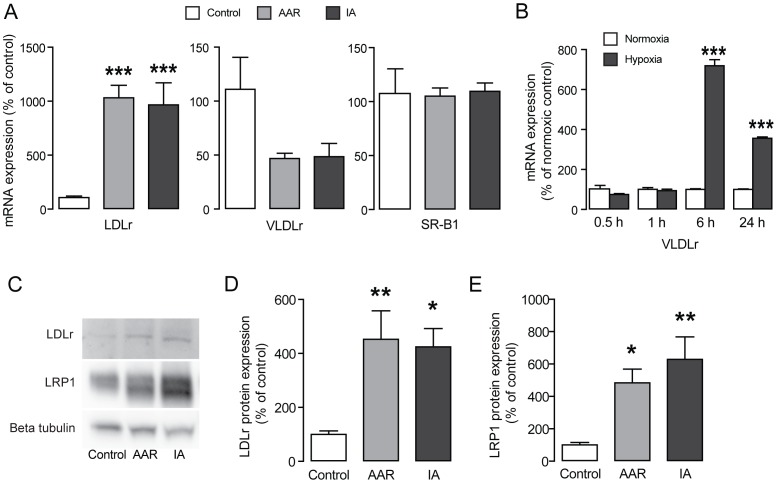
Increased expression of LDLr and LRP1. **(A)** RT-QPCR analyses of mRNA expression of LDLr, VLDLr and SR-B1, n = 4 per group. **(B)** mRNA expression of VLDLr in HL-1 cells incubated in hypoxia for 0.5, 1, 6 and 24 h (n = 3). **(C)** Representative immunoblots of LDLr and LRP1. (D–E) Quantification of LDLr protein bands **(D)** and LRP1 protein bands **(E)** n = 6–7 per group. Results are shown as mean ± SEM, *P<0.05 *vs.* control, **P<0.01 *vs.* control, ***P<0.001 *vs.* control.

Cholesteryl esters are taken up by cells via LDL receptor (LDLr) mediated endocytosis. Therefore, we measured the mRNA level of the LDLr in control tissue, AAR and IA. Indeed, LDLr mRNA expression was increased by 10-fold in AAR as well as IA compared with control (Figure 3A), while expression of VLDLr and scavenger receptor B1 (SR-B1) not was increased (Figure 3A). Because we have previously seen that the VLDLr is up-regulated by hypoxia in the mouse heart, [3] we wanted to investigate the hypoxic time period required to up-regulate the VLDLr. We found that the VLDLr was not was increased after shorter periods of hypoxia but up-regulated only first after 6 hours in hypoxia (Figure 3B). Furthermore, we found that protein levels of the LDLr in the ischemic pig heart were increased by more than 4-fold (Figure 3C and 3D). Interestingly, we also found increased protein expression of low density lipoprotein receptor-related protein 1 (LRP1) (Figure 3C and 3E).

### Myocardial ischemia promotes accumulation of ceramides in the infarct area

**Figure 4:**
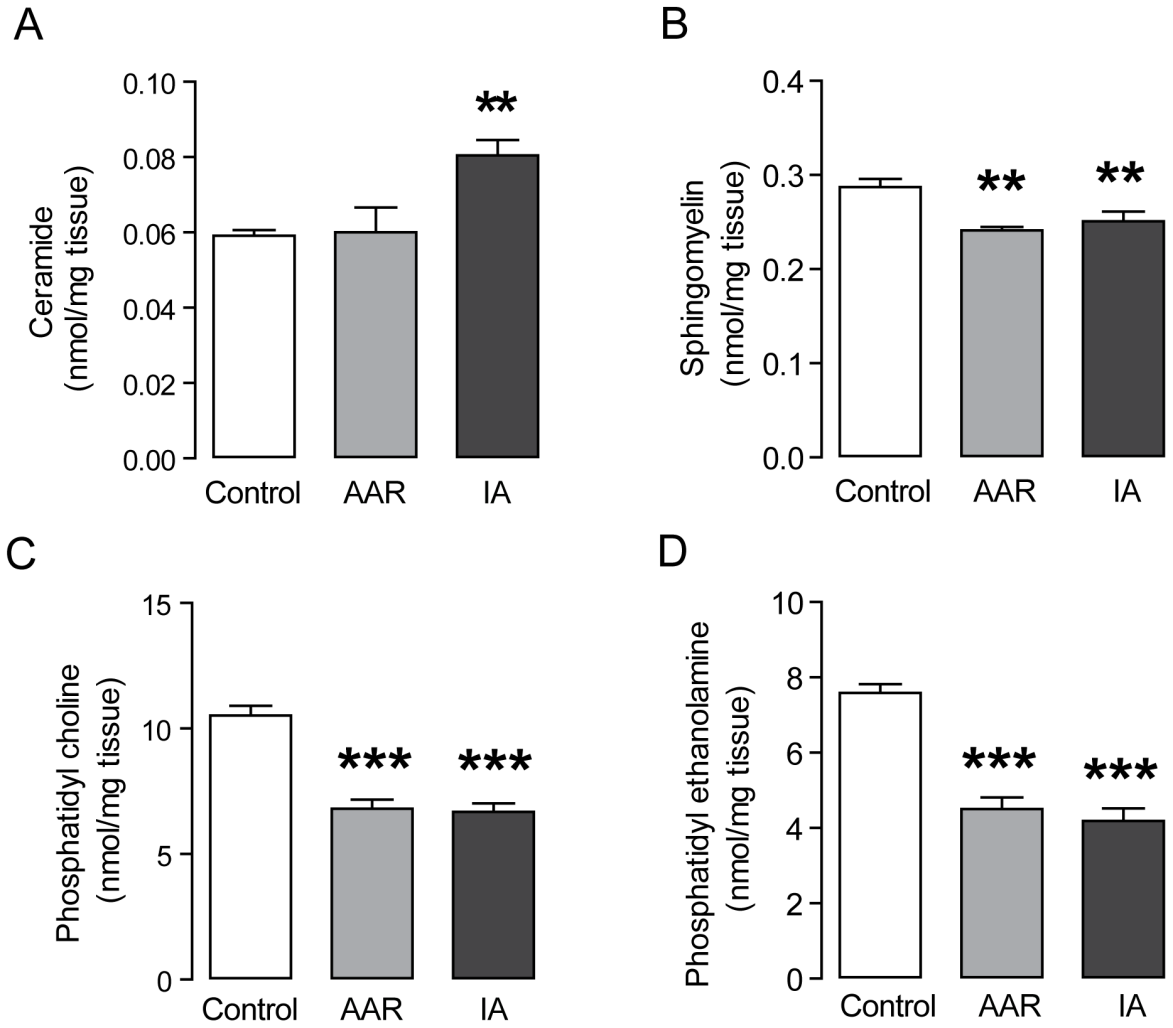
Increased levels of ceramides in ischemic myocardium. Content of ceramide (A), sphingomyelin (B), phosphatidylcholine (C) and phosphatidylethanolamine (D), n = 7 per group. Results are shown as mean ± SEM, **P<0.01 *vs.* control, ***P<0.001 *vs.* control.

Increased levels of neutral lipids in the heart are not per se believed to be detrimental for heart function. Rather, levels of toxic lipid metabolites, such as the ceramides, accumulate under conditions of extreme lipid loading and cause cellular dysfunction. [19], [20] We therefore wanted to assess the levels of accumulating sphingo- and phospholipids. Interestingly, we found that ceramide levels were increased in the IA but not in the AAR (Figure 4A), suggesting that ceramide levels are normalized when the viable heart tissue is reperfused but that they accumulate in the infarcted area with irreversible damage. Furthermore, we found that sphingomyelin levels were decreased in AAR as well as IA compared with control tissue (Figure 4B). The phospholipids phosphatidyl choline and phosphatidyl ethanolamine were both decreased in AAR and IA (Figure 4C and D).

### Ischemia-induced inflammation and ER stress are not reversed by reperfusion

**Figure 5:**
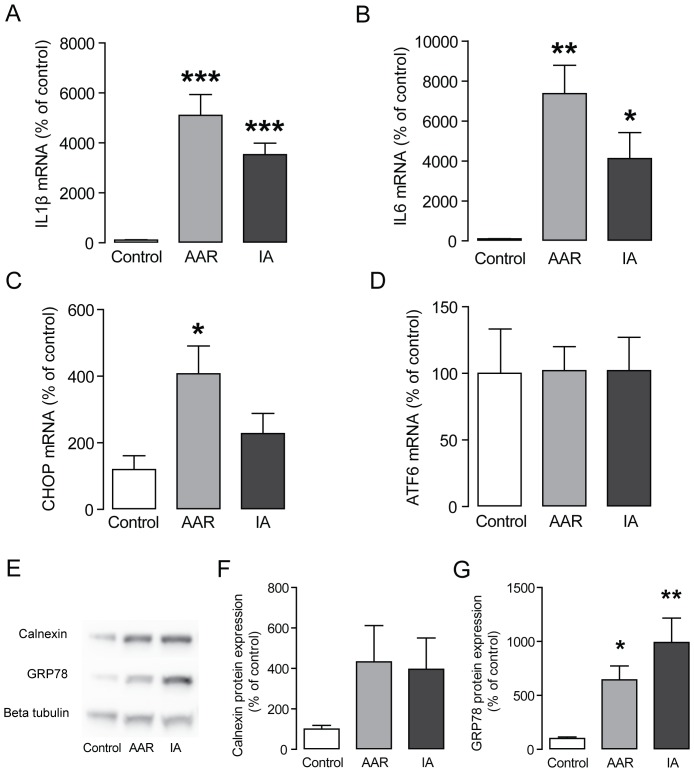
Increased expression of the cytokines IL-1β and IL-6 and ER stress markers in ischemic myocardium. (**A**–**B**) RT-QPCR analyses of mRNA expression of IL-1β (**A**) and IL-6 (**B**), n = 6 per group. (**C**–**D**) RT-QPCR analyses of mRNA expression of CHOP (C) and ATF6B (**D**), n = 6 per group. (E) Representative immunoblots of Calnexin and GRP78. (**F**–**G**) Quantification of LDLr protein bands (F) and LRP1 protein bands (G) n = 7 per group. Results are shown as mean ± SEM, *P<0.05 *vs.* control, **P<0.01 *vs.* control, ***P<0.001 *vs.* control.

Myocardial ischemia and reperfusion is accompanied with a marked inflammatory reaction. [21], [22] In agreement, we show a striking up-regulation of the cytokines IL-1β and IL-6 in the IA and the AAR compared with control area (Figure 5A–B). Interestingly, this inflammatory response is not lower in the viable AAR, but rather more increased (Figure 5A–B). Lipid accumulation and/or inflammation have in various tissues been reported to induce ER stress. [23] To investigate if myocardial ischemia promotes ER stress in our model, we analyzed if ER stress markers CHOP, ATF6B, calnexin and GRP78 were altered. We found that the expression of CHOP increased in the AAR, but it was surprisingly not significantly up-regulated in the IA (Figure 5C). Expression of ATF6B was unchanged in the AAR as well as the IA (Figure 5D). In addition, protein expression of calnexin and GRP78 was increased in the AAR and in the IA (Figures 5E–G). Hence, inflammation as well as ER stress is activated in the AAR and in the IA.

## Discussion

In this study, we investigated whether myocardial ischemia promotes lipid accumulation in a porcine model. We found that myocardial ischemia promoted accumulation of cholesteryl esters in the pig heart and that this accumulation was mediated via increased expression of the LDLr and LRP1. Furthermore, we found increased ceramide accumulation and inflammation in the infarcted area of the pig heart. In addition, we found increased levels of cholesteryl esters, inflammation and ER stress in the myocardium bordering the infarct area.

We have previously shown that an acute myocardial infarction in a mouse model promotes accumulation of triglycerides, mediated by an increased expression of the VLDLr. [3] In contrast, we did not find accumulation of triglycerides in the pig heart after ischemia, but instead we found greatly increased levels of cholesteryl esters. We also found that the expression of the LDLr and LRP1 was up-regulated, indicating that cholesteryl ester accumulation is mediated via uptake through the LDLr and LRP1, and not through the VLDLr. It is possible that there are species differences regarding which lipids that accumulate after myocardial ischemia. Alternatively, the explanation may be differences in time. In our previous study in mice, myocardial ischemia was induced for 8–24 hours and in this study, myocardial ischemia was induced for 40 min following 4 hours of reperfusion. Indeed, a time study conducted in HL-1 cells showed an increase of the VLDLr first after 6 hours of incubation in hypoxia. Thus, it is conceivable that expression of the VLDLr and triglyceride uptake occurs after a longer time period of ischemia. We have previously presented data showing that the expression of VLDLr is responsive to HIF-1α. We therefore analyzed the expression of HIF-1α responsive genes in the infarcted pig hearts (data not shown) and found increases in some HIF-1α responsive genes but not all investigated. Our results suggest that the short ischemia period result in a very modest up-regulation of HIF-1α, which may explain the absence of the HIF-1α associated up-regulation of VLDLr in the pig ischemia/reperfusion model. An additional difference between this porcine model and previous studies conducted in mice is the choice of control. In the porcine model, the control area was a non-ischemic biopsy from each heart, whereas in the mouse model, sham operated hearts were used as controls. Hence, the control area in the pig heart was located in a heart severely injured by an infarction as opposed to the uninjured sham operated heart controls. Thus, the gene expression pattern may differ between these controls, which might affect the regulation of the VLDLr. Cholesteryl esters have previously been reported to accumulate after induced hypoxia in rat cardiomyocytes. [24] However, the uptake of CE was in that model mediated by LRP1 and the VLDLr. [24] In this ischemia/reperfusion model, we found an up-regulation of LDLr as well as LRP1. It is possible that uptake of cholesteryl esters in the porcine model is mediated by LRP1 and that the LDLr is up-regulated by the inflammatory response. Indeed, it has previously been shown that inflammatory stress (e.g. IL-1β) up-regulates LDLr-mediated cholesterol influx by increasing the transcription of LDLr [14], [15].

Accumulation of lipids in the myocardium has been shown to associate with inflammation, ER stress, apoptosis and reduced heart function. [4], [7] For example, high levels of cholesteryl esters have been reported to associate with increased myocardial oxidative stress, inflammation and apoptosis in ischemic heart. [25] In addition, it has been reported that there is a close association between cholesteryl ester content of sarcoplasmatic reticulum and sarcoplasmic/endoplasmic reticulum Ca2+-ATPase (SERCA-2) suppression. [26] In addition, accumulation of bioactive lipids, such as sphingolipids, has been proposed to exert lipotoxic effects on the myocardium. [19] For instance, ceramides have been shown to induce apoptosis following a myocardial infarction (reviewed in [19]). In our model, we found that ceramides were increased in IA, but not altered in the AAR. Our results suggest that ceramide levels are normalized when the viable heart tissue is reperfused but that they accumulate in the infarcted area with irreversible damage. Inhibition of de novo ceramide synthesis in mice with lipotoxic cardiac dysfunction has been shown to improve heart function, suggesting that accumulating ceramides are synthesized de novo. However, ceramides may well be originating from sphingomyelin breakdown. Further investigation will be needed to elucidate which pathway that is activated due to myocardial ischemia.

Conditions of the ER must be optimal for proper synthesis and folding of proteins. Ischemia has been suggested to activate stress of the ER. [27], [28] ER stress will initiate the unfolded protein response (UPR), which aims at restoring the ER environment. However, if the stress continues and ER protein folding is not re-established, ER stress may lead to apoptosis. [29] In our model, ER stress was activated in the area at risk as well as in the IA, suggesting that the UPR is stimulated due to the myocardial ischemia. The surviving myocardium bordering the infarct is exposed to mild ischemia and is stressed, but remains viable. Improving function of the myocardium bordering the infarct would therefore be the optimal target for therapeutics. Reperfusion of the damaged myocardium has proven to be the most efficient approach to reduce infarct size. However, reperfusion of the myocardium may paradoxically include detrimental effects to the tissue, referred to as reperfusion injury. Reperfusion injury can activate a smaller but continuous wave of cell death, as well as changes in the metabolic and functional characteristics of surviving cells. Indeed, in our model inflammation and ER stress is more activated in the AAR than the IA, indicating that reperfusion per se results in inflammation and ER stress.

In conclusion, we found that myocardial ischemia promoted accumulation of cholesteryl esters and ceramides in the infarct area in the pig heart and that this accumulation was mediated via increased expression of the LDLr and LRP1. In addition, we found increased levels of cholesteryl esters, inflammation and ER stress in the myocardium bordering the infarct area. Our results indicate that lipid accumulation in the heart is one of the metabolic derangements remaining after ischemia, even in the peri-infarct myocardium. Normalizing lipid levels in the myocardium after ischemia would likely improve myocardial function and should therefore be considered as a target for treatment.
